# Metabolomic approach to the exploration of biomarkers associated with disease activity in rheumatoid arthritis

**DOI:** 10.1371/journal.pone.0219400

**Published:** 2019-07-11

**Authors:** Chiyomi Sasaki, Tomoko Hiraishi, Takuma Oku, Kenji Okuma, Kenichi Suzumura, Motomu Hashimoto, Hiromu Ito, Ichiro Aramori, Yoshitaka Hirayama

**Affiliations:** 1 Center for Innovation in Immunoregulative Technology and Therapeutics, Graduate School of Medicine, Kyoto University, Kyoto, Kyoto, Japan; 2 Candidate Discovery Science Labs, Astellas Pharma Inc., Tsukuba, Ibaraki, Japan; 3 Analysis & Pharmacokinetics Research Labs., Astellas Pharma Inc., Tsukuba, Ibaraki, Japan; 4 Department of Advanced Medicine for Rheumatic Diseases, Graduate School of Medicine, Kyoto University, Kyoto, Kyoto, Japan; 5 Department of Orthopedic Surgery, Graduate School of Medicine, Kyoto University, Kyoto, Kyoto, Japan; Indian Institute of Chemical Technology, INDIA

## Abstract

We aimed to investigate metabolites associated with the 28-joint disease activity score based on erythrocyte sedimentation rate (DAS28-ESR) in patients with rheumatoid arthritis (RA) using capillary electrophoresis quadrupole time-of-flight mass spectrometry. Plasma and urine samples were collected from 32 patients with active RA (DAS28-ESR≥3.2) and 17 with inactive RA (DAS28-ESR<3.2). We found 15 metabolites in plasma and 20 metabolites in urine which showed a significant but weak positive or negative correlation with DAS28-ESR. When metabolites between active and inactive patients were compared, 9 metabolites in plasma and 15 in urine were found to be significantly different. Consequently, we selected 11 metabolites in plasma and urine as biomarker candidates which significantly correlated positively or negatively with DAS28-ESR, and significantly differed between active and inactive patients. When a multiple logistic regression model was built to discriminate active and inactive cohorts, three variables—histidine and guanidoacetic acid from plasma and hypotaurine from urine—generated a high area under the receiver operating characteristic (ROC) curve value (AUC = 0.8934). Thus, this metabolomics approach appeared to be useful for investigating biomarkers of RA. Combination of plasma and urine analysis may lead to more precise and reliable understanding of the disease condition. We also considered the pathophysiological significance of the found biomarker candidates.

## Introduction

Rheumatoid arthritis (RA) is a systemic autoimmune disease which involves inflammation of the synovium and destruction of joint cartilage and bone [1,2]. RA is pathologically heterogeneous, with many suspected triggers for development of the disease, including environmental [3], epigenetic [4], and genetic factors [5–7] as well as several types of post-translational modifications of proteins [2]. The complexity of the disease is further suggested by the various clinical features of RA, as well as the differences in response to therapies among patients treated with synthetic and/or biological disease modifying anti-rheumatic drugs (DMARDs) [2,8,9].

To date, various omics studies have aimed to better understand the molecular pathophysiology of RA and explore the disease condition in individual patients. In recent years, metabolomics has been acknowledged to be a powerful tool for identifying potential biomarkers in RA patients using different types of samples such as plasma, serum, urine, and synovial fluids [10–14]. The advantages of metabolomics may not only be in the discovery of biomarkers but also in the identification of rapid physiological responses according to disease activities, as well as in evaluation of the prognosis and therapeutic response to treatment and understanding the pathophysiology of the disease. However, the correlation of the dynamics of metabolites with the disease activity of RA has not been well investigated.

In this study, we obtained urine and plasma samples from biologics-naive RA patients, and searched for metabolites associated with disease activity using capillary electrophoresis quadrupole time-of-flight mass spectrometry (CE-Q-TOFMS). This method allows almost any polar and charged species to be analyzed, combines high-resolution separations with high detection selectivity and sensitivity, and maintains high reproducibility [15,16].

## Materials and methods

### Study cohorts

The study protocol was approved by the Ethics Committee, Kyoto University Graduate School and Faculty of Medicine. We collected blood and urine from 50 RA patients diagnosed with RA based on the American College of Rheumatology guidelines at the Rheumatic Disease Center, Kyoto University Hospital. The data of one male patient was omitted because he was receiving hemodialysis. No patient had received treatment with biologics, and RA disease activity was categorized based on the 28-joint disease activity score based on erythrocyte sedimentation rate (DAS28-ESR). Patients with DAS28-ESR≥3.2 and those with DAS28-ESR<3.2 were defined as active and inactive patients, respectively. Other clinical information was obtained from the medical records. Blood was collected from 10 non-RA volunteers matched for age and gender who served as controls. All RA patients and control subjects were recruited from November 2012 to May 2013, and written informed consent was obtained from all participants on the day of sampling.

### Sample preparation

All blood and urine samples were kept at 4°C immediately after collection and processed within 1 hour. Plasma were prepared from EDTA-anticoagulated blood. All plasma and urine samples were aliquoted and stored at -80°C until further analysis.

### Metabolomics analysis

Plasma or urine samples (50 μL) were added to 450 μL of methanol (134–14523, FUJIFILM Wako Pure Chemical Corporation [Wako], Osaka, Japan) containing internal standards (H3304-1002; Human Metabolome Technologies, Inc. [HMT], Tsuruoka, Japan), 200 μL of Milli-Q water and 500 μL of chloroform (033–08631, Wako). The samples were then thoroughly mixed by vortex mixer and centrifuged at 9,100 × g at 4°C for 20 min. Subsequently, 350 μL of the upper aqueous layer was centrifugally filtered through a 5-kDa cutoff filter (provided by HMT) at 9,100 × g overnight at 4°C to remove proteins and macromolecules. The filtrate was evaporated and resuspended in 50 μL of Milli-Q water containing internal standards (H3304-1004, HMT) for CE-Q-TOFMS.

A Capillary Electrophoresis System (Agilent Technologies, Santa Clara, California) with an Agilent 6510 Q-TOF mass spectrometer (Agilent Technologies) was used for CE-Q-TOFMS. The fused silica capillary and analysis reagents were provided by HMT. To analyze cationic metabolites, the sample solution was injected at a pressure of 50 mbar for 10 s, and the applied voltage was set at 27 kV. Capillary and fragmenter voltage in positive ion mode were set at 4000 and 80 V. A flow rate of heated dry N2 gas (heater temperature, 300°C) was maintained at 5 psig and 7 L/min. The spectrometer was scanned from *m/z* 100 to 3000. To analyze anionic metabolites, the sample solution was injected at a pressure of 50 mbar for 25 s, and the applied voltage was 30 kV. Capillary and fragmenter voltage in negative ion mode were set at 3500 and 125 V. A flow rate of heated dry N2 gas (heater temperature, 300°C) was maintained at 5 psig and 7 L/min. The spectrometer was scanned from *m/z* 100 to 3000. Other conditions were as described previously [17], with slight modifications.

Data processing of MS was started by extracting peaks using MasterHands automatic integration software (Keio University, Tsuruoka, Japan) to obtain peak information, including *m/z*, migration time (MT), and peak area [18]. Signal peaks corresponding to isotopomers, adduct ions, and other product ions of known metabolites were excluded, and remaining peaks were annotated with putative metabolites from the MasterHands database based on their MTs and *m/z* values. The tolerance range for the peak annotation was configured at ±0.2 min (Anion)/±1.0min (Cation) for MT and ±40 ppm for *m/z*. In addition, peak areas were normalized against those of the internal standards, and relative area values of urine samples were further normalized by creatinine ^13^C peak. The metabolite IDs were adopted from the Kyoto Encyclopedia of Genes and Genomes database (KEGG, https://www.genome.jp/kegg/).

### Statistical analysis

Student’s t test or Welch’s t test was performed to assess statistical significance of differences between the two groups using Genedata Analyst (Genedata AG., Basel, Switzerland). Fisher’s exact test was performed to assess categorical variables with JMP Pro 12.2.0 (SAS Institute Inc., Cary, NC, USA). Correlation of metabolites with DAS28-ESR was analyzed by the Spearman rank correlation test with JMP Pro. Principal component analysis (PCA), partial least-squares discriminant analysis (PLS-DA) and validation of the PLS-DA model by permutation tests were conducted with normalize metabolomics data using MetaboAnalyst 4.0 (ref [19], http://www.metaboanalyst.ca/). A multiple logistic regression (MLR) model to discriminate active and inactive cohorts was developed by a stepwise variable selection method (forward and backward selection), conducted with a threshold of p<0.1 for adding and eliminating features with JMP Pro.

## Results

### Subject characteristics

The primary characteristics of the RA patients and control subjects are shown in Table 1. We recruited 32 active (DAS28-ESR≥3.2) and 17 inactive (DAS28-ESR<3.2) RA patients. Most RA patients had been treated with methotrexates and/or glucocorticoids, and none had been treated with biologics.

**Table 1:** Profiles of control subjects and RA patients.

	Control	All RA	P-value^2)^	Active RA^1)^	Inactive RA^1)^	P-value^3)^
Number	10	49		32	17	
Age	63 ± 14	60 ± 13	0.540	61 ± 13	59 ± 12	0.492
(range)	(51–86)	(34–81)		(34–81)	(34–81)	
Sex ratio	10/0	43/6	0.577	27/5	16/1	0.650
(female/male)						
DAS28-ESR	-	3.71 ± 1.23		4.38 ± 0.94	2.46 ± 0.54	<0.001
(range)		(1.12–7.62)		(3.23–7.62)	(1.12–3.11)	
Treatment	-	MTX: 39		MTX: 27	MTX: 12	0.285
		GCs: 22		GCs: 17	GCs: 5	0.140

RA, rheumatoid arthritis; DAS28-ESR, disease activity score using 28 joint counts based on erythrocyte sedimentation rate; MTX, methotrexate; GCs, glucocorticoids.

^1)^ Active patients and inactive patients was defined as patients with DAS28-ESR≥3.2 and those with DAS28-ESR<3.2, respectively.

^2)^ Student’s t test or Fisher’s exact test between control and RA groups.

^3)^ Student’s t test or Fisher’s exact test between active and inactive RA groups.

Values are expressed as mean ± standard deviation (SD) and ranges (minimum to maximum).

### Comparison of metabolites in plasma between RA patients and control subjects

Using the CE-Q-TOFMS method, 104 metabolites in plasma and 217 metabolites in urine were identified and quantified (S1 Dataset). Since ketoprofen found in urine was an exogenous metabolite, we excluded it from the following analysis.

**Fig 1:**
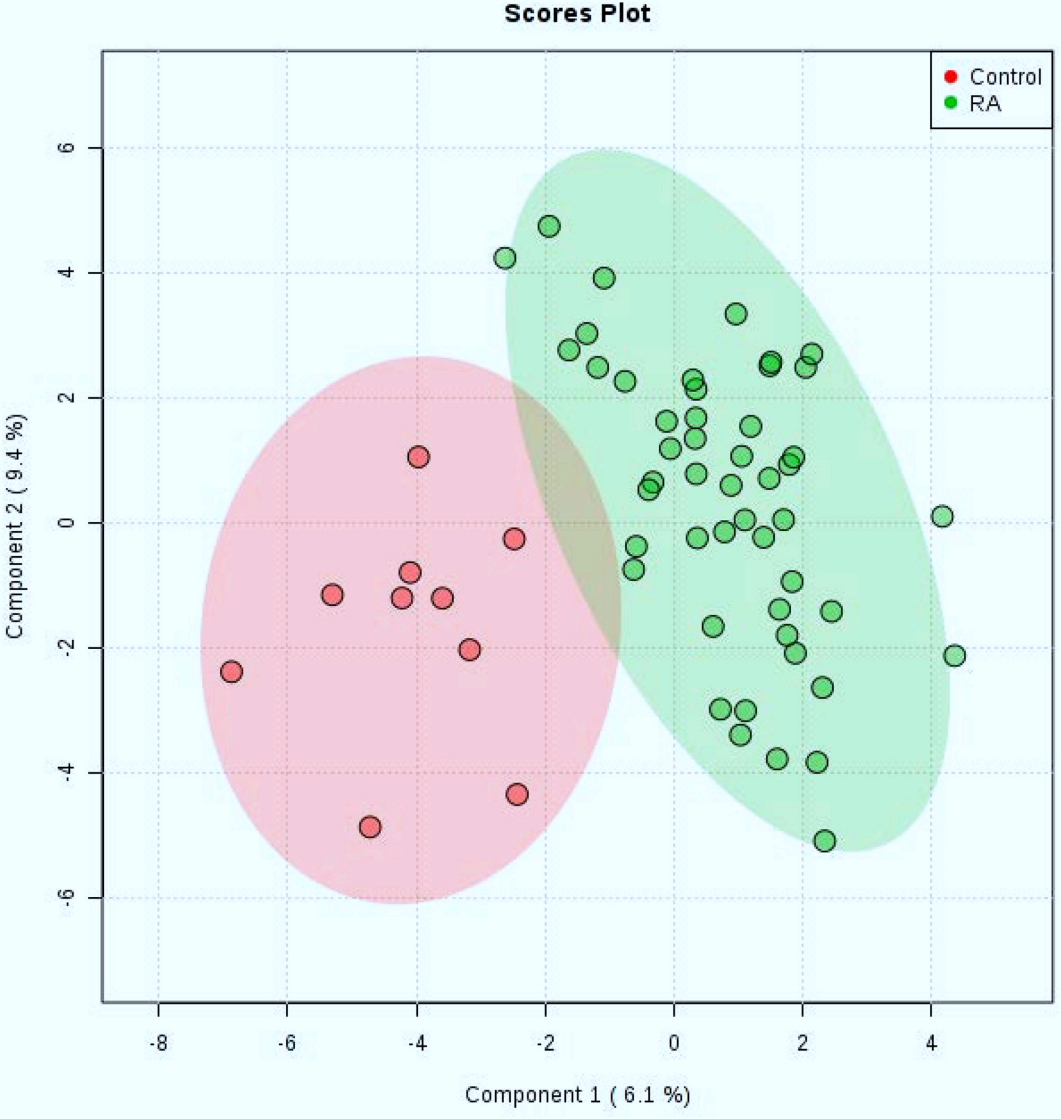
PLS-DA score plot between RA patients (n = 49) and control subjects (n = 10) based on metabolic profiles in plasma. The green and red dots represent RA patient and control samples, respectively.

First, to evaluate the validity of the collected patient samples, principal component analysis (PCA) was performed using plasma metabolites from RA patients and control subjects, but the results showed no solid separation between the two groups (S1 Fig). However, when PLS-DA was performed, results demonstrated an acceptable cluster between the two groups (Fig 1) with good model parameters (R^2^ = 0.75529, Q^2^ = 0.4068). Validation of the PLS-DA model by permutation tests showed p = 0.022 (S2 Fig), which indicated that the separation was significant.

When the metabolites from RA patients and controls were compared, we found 24 metabolites that were significantly different (Welch t-test with p<0.05) between the two groups (Table 2). Some of the metabolites were in agreement with previously published data that compared RA patients and control subjects, such as decreased levels of histidine, methionine, and serine, and increased levels of glyceric acid, phenylalanine, and tyrosine in RA patients [12,14,20–22]. Moreover, the identified metabolites were major intermediates of metabolic pathways, including glycolysis, the tricarboxylic acid (TCA) cycle, and pathways involving amino acid metabolism, which were also in agreement with previous reports [12,14]. These data suggest that the collected samples were not derived from exceptional RA patients.

**Table 2:** Metabolites in plasma that were significantly different between RA patients and control subjects.

Metabolite	KEGG ID	Mode	*m/z*	MT	P-value^1)^	Fold change^2)^
						RA/Control
Azelaic acid	C08261	A	187.097	12.583	<0.001	-2.98
N-Acetylleucine	C02710	A	172.098	8.229	<0.001	-2.21
Pyruvic acid	C00022	A	87.009	13.462	<0.001	1.99
Phenylalanine	C00079	C	166.087	12.137	<0.001	1.36
Glycerol-3-phosphate	C00093	A	171.006	12.623	0.001	1.89
Cysteine-glutathione disulphide	N/A	C	427.096	12.759	0.002	-1.69
Glutamic acid; threo-beta-methylaspartic acid	C00025; N/A	C	148.061	11.953	0.002	1.44
Glyceric acid	C00258	A	105.019	10.814	0.002	1.26
Tyrosine	C00082	C	182.082	12.438	0.005	1.19
Cysteine-glutathione disulphide–Divalent	N/A	C	214.052	12.757	0.005	-1.56
Glucuronic acid; Galacturonic acid	C00191; C08348	A	193.035	8.302	0.006	1.99
3-Methylhistidine	C01152	C	170.093	8.046	0.007	1.70
Gluconic acid	C00257	A	195.051	8.344	0.017	1.25
Threonic acid	C01620	A	135.030	9.518	0.020	1.32
Pelargonic acid	C01601	A	157.123	8.331	0.023	1.14
gamma-Butyrobetaine	C01181	C	146.118	8.714	0.024	-1.34
Asymmetric dimethylarginine	C03626	C	203.149	8.251	0.026	1.11
Serine	C00065	C	106.050	10.844	0.028	-1.19
Histidine	C00135	C	156.077	7.824	0.029	-1.11
N,N-Dimethylglycine	C01026	C	104.071	11.945	0.032	1.25
1-Methylnicotinamide	C02918	C	137.069	7.882	0.037	-1.57
Mucic acid; Glucaric acid	C00879; C00818	A	209.030	14.658	0.039	1.77
Lactic acid	C00186	A	89.025	11.226	0.043	1.23
2-Hydroxybutyric acid; 2-Hydroxyisobutyric acid	C05984; N/A	A	103.040	10.084	0.049	1.22

A, anion mode; C, cation mode; MT, migration time; N/A, not applicable

^1)^ P-values are calculated by Welch’s t test between RA patients and control subjects.

^2)^ Fold changes are shown as ratio of mean value of RA patients versus that of control subjects. If the number was less than one, the negative value is shown.

### Metabolites associated with DAS28-ESR

Next, we sought biomarkers that were associated with RA disease activity. We performed PCA between active (DAS28-ESR≥3.2) and inactive (DAS28-ESR<3.2) patients based on metabolic profiles in plasma and urine, but no solid separation was seen (S3 Fig). PLS-DA apparently showed a clear separation, but the result suggested overfitting (S4 Fig). Thus, we decided to search for metabolites that significantly correlated with DAS28-ESR. As a result, we found 7 and 8 metabolites that positively and negatively correlated with DAS28-ESR, respectively, in patient plasma samples (Table 3), and 16 and 4 in urine, respectively. There were no overlapping metabolites in both plasma and urine.

**Table 3:** Metabolites in plasma and urine of RA patients which significantly correlated with DAS28–ESR.

Metabolite	KEGG ID	Mode	*m/z*	MT	Spearman ρ	P-value
***Plasma***						
Glucuronic acid; Galacturonic acid	C00191; C08348	A	193.035	8.302	0.378	0.007
Urea	C00086	C	61.041	24.252	0.376	0.008
N,N-Dimethylglycine	C01026	C	104.071	11.945	0.365	0.010
Gluconic acid	C00257	A	195.051	8.344	0.354	0.013
Cysteine	C00097	C	122.027	12.045	0.298	0.038
Sarcosine	C00213	C	90.055	10.268	0.292	0.042
3-Methylhistidine	C01152	C	170.093	8.046	0.287	0.046
4-Methyl-2-oxopentanoic acid; 3-Methyl-2-oxovaleric acid	C00233; C03465	A	129.055	9.865	-0.298	0.038
Cysteine-glutathione disulphide	N/A	C	427.096	12.759	-0.306	0.033
Homoarginine; N6,N6,N6-Trimethyllysine	C01924; C03793	C	189.141	7.718	-0.318	0.026
Cysteine-glutathione disulphide -Divalent	N/A	C	214.052	12.757	-0.323	0.023
Citric acid	C00158	A	191.020	27.938	-0.324	0.023
Methionine	C00073	C	150.059	11.709	-0.361	0.011
Guanidoacetic acid	C00581	C	118.062	8.874	-0.400	0.005
Histidine	C00135	C	156.077	7.824	-0.477	0.001
***Urine***						
2-Quinolinecarboxylic acid	C06325	A	172.044	9.215	0.378	0.008
4-Hydroxy-3-methoxymandelic acid; Syringic acid	C05584; C10833	A	197.047	8.349	0.360	0.011
N-Acetylneuraminic acid	C00270	A	308.099	7.282	0.350	0.014
p-Hydroxyphenylacetic acid; p-Anisic acid	C00642; C02519	A	151.040	8.898	0.340	0.017
Homoserine	C00263	C	120.064	10.947	0.325	0.023
Riboflavin	C00255	C	377.135	25.500	0.322	0.026
2'-Deoxycytidine	C00881	C	228.090	10.184	0.319	0.026
Gibberellic acid	C01699	A	345.153	7.101	0.318	0.026
1-Methyl-4-phenyl-1,2,3,6-tetrahydropyridine	C04599	C	174.124	9.769	0.311	0.030
gamma-Glu-2-aminobutanoic acid	N/A	C	233.113	13.643	0.307	0.032
Methylguanidine	C02294	C	74.071	6.578	0.306	0.033
3-Hydroxy-3-methylglutaric acid	C03761	A	161.045	16.032	0.302	0.035
Hypotaurine	C00519	C	110.027	20.735	0.298	0.038
N-Acetylglucosamine 1-phosphate	C04256	A	300.041	9.910	0.285	0.047
4-Oxovaleric acid	N/A	A	115.040	9.912	0.284	0.048
Threonic acid	C01620	A	135.030	9.479	0.284	0.048
N6,N6,N6-Trimethyllysine	C03793	C	189.160	7.636	-0.283	0.049
Hypoxanthine	C00262	C	137.046	12.041	-0.304	0.034
gamma-Butyrobetaine	C01181	C	146.118	8.695	-0.304	0.034
Alanine	C00041	C	90.056	9.758	-0.310	0.030

A, anion mode; C, cation mode; MT, migration time; N/A, not applicable

Further, we compared metabolites between active and inactive patients. As shown in Table 4, 9 metabolites in plasma and 15 metabolites in urine were identified to be significantly different (Welch t-test with p<0.05) between active and inactive RA patients. Again, there were no metabolites that were detected both in plasma and urine.

**Table 4:** Metabolites in plasma and urine that were significantly different between active RA patients and inactive RA patients.

Metabolite	KEGG ID	Mode	*m/z*	MT	P-value^1)^	Fold change^2)^
						Active/inactive
***Plasma***						
Histidine	C00135	C	156.077	7.824	0.003	-1.13
Urea	C00086	C	61.041	24.252	0.004	1.26
N,N-Dimethylglycine	C01026	C	104.071	11.945	0.007	1.33
Guanidoacetic acid	C00581	C	118.062	8.874	0.010	-1.30
Homoarginine; N6,N6,N6-Trimethyllysine	C01924; C03793	C	189.141	7.718	0.011	-1.24
3-Phenylpropionic acid	C05629	A	149.059	8.998	0.022	1.44
Phenylalanine	C00079	C	166.087	12.137	0.024	1.27
3-Indoxylsulfuric acid	N/A	A	212.002	9.883	0.031	1.77
beta-Alanine	C00099	C	90.055	7.868	0.049	1.27
***Urine***						
2-Quinolinecarboxylic acid	C06325	A	172.044	9.215	0.002	3.85
Gibberellic acid	C01699	A	345.153	7.101	0.002	3.52
Riboflavin	C00255	C	377.135	25.500	0.006	9.95
N-Acetylglucosamine 1-phosphate	C04256	A	300.041	9.910	0.009	3.10
3-Indoxylsulfuric acid	N/A	A	212.003	9.821	0.013	1.74
m-Hydroxybenzoic acid	C00587	A	137.023	9.555	0.013	2.50
5-Methoxyindoleacetic acid	C05660	C	206.077	25.628	0.017	2.76
Hypotaurine	C00519	C	110.027	20.735	0.017	1.59
Anserine; Homocarnosine	C01262; C00884	C	241.130	7.354	0.023	2.60
4-Guanidinobutyric acid	C01035	C	146.094	8.892	0.023	1.48
Ophthalmic acid	N/A	C	290.135	14.578	0.024	1.48
Azetidine 2-carboxylic acid	C08267	C	102.055	9.568	0.030	1.72
2,6-Diaminoheptanedioic acid	C00666	C	191.102	9.574	0.036	3.09
Betonicine	C08269	C	160.097	14.514	0.038	4.12
1-Methyl-4-phenyl-1,2,3,6-tetrahydropyridine	C04599	C	174.124	9.769	0.039	2.08

A, anion mode; C, cation mode; MT, migration time; N/A, not applicable

^1)^ P-values are calculated by Welch’s t test between active and inactive RA patients.

^2)^ Fold changes are shown as ratio of mean value of active RA patients versus that of inactive RA patients. If the number was less than one, the negative value is shown.

Consequently, we selected 11 metabolites as biomarker candidates, which significantly correlated with DAS28-ESR, either positively or negatively, as well as those that were significantly different between active and inactive patients. The 11 metabolites were as follows: guanidoacetic acid, histidine, homoarginine or N6,N6,N6-trimethyllysine, N,N-dimethylglycine, and urea in plasma, 1-methyl-4-phenyl-1,2,3,6-tetrahydropyridine, 2-quinolinecarboxylic acid, gibberellic acid, hypotaurine, N-acetylglucosamine 1-phosphate, and riboflavin in urine.

### MLR analysis

**Fig 2:**
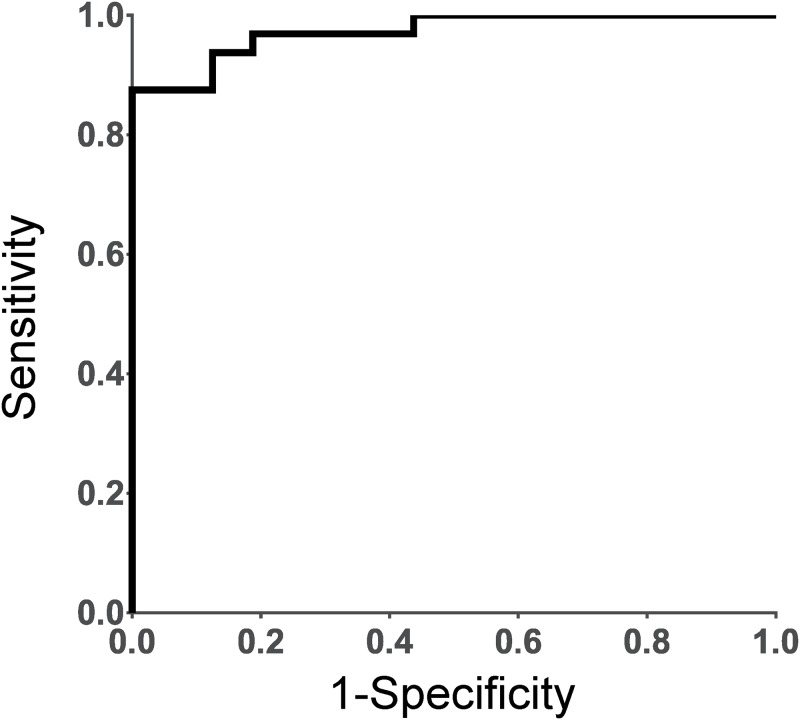
ROC curve of the metabolites that correlated with DAS28-ESR and significantly differed between active and inactive patients. The selected metabolites in this model were histidine and guanidoacetic acid in plasma and hypotaurine in urine.

We developed MLR model to search for potential biomarkers of RA disease activity. First, we selected three metabolites, histidine and guanidoacetic acid in plasma and hypotaurine in urine, as MLR variables, which were metabolites that both correlated significantly with DAS28-ESR and significantly differed between active and inactive patients, by stepwise feature selection. Taking these three factors, the model yielded a high value of area under the receiver operating characteristic (ROC) curve (AUC = 0.8934), as shown in Fig 2. This result indicated that combining plasma and urine metabolomics analysis also identified biomarkers that correlated closely with the disease activity of RA patients.

## Discussion

In this study, we found several candidate biomarkers of RA disease activity from metabolites in plasma and urine by the CE-Q-TOFMS method. Interestingly, only a few common metabolites were found in plasma and urine, which implied that different biomarkers could be found from the two biofluids. Indeed, we identified two metabolites in plasma, histidine and guanidoacetic acid, and one metabolite, hypotaurine, in urine, as possible biomarkers that may be closely associated with RA disease activity.

As in our study, low histidine concentration has been previously reported in RA [20–22], as well as in other diseases, such as chronic kidney disease and gallbladder inflammation with chronic cholecystitis [23, 24]. Since histidine is considered to be an anti-inflammatory and antioxidant factor [25, 26], it may be associated with the inflammation state. However, we found no other metabolites in histidine-related metabolic pathways that are associated with RA disease activity. Thus, further investigation is needed to find the underlying mechanism of how histidine level decreases in RA patients.

Guanidoacetic acid is involved in the arginine metabolism pathway. It is synthesized by the enzyme arginine:glycine amidinotransferase (AGAT) from arginine or glycine. Homoarginine is also synthesized by AGAT from arginine or lysine [27], and both guanidoacetic acid and homoarginine were inversely correlated with DAS28-ESR and decreased in active RA patients (Tables 3 and 4). In the pathway, guanidoacetic acid is then synthesized into creatine by guanidoacetic acid N-methyltransferase, which is subsequently catalyzed by creatinase to produce urea and sarcosine, both of which correlated significantly with DAS28-ESR in plasma (Table 3). These data suggest that the metabolism of arginine/glycine/lysine-guanidoacetic acid/homoarginine-urea/sarcosine pathway may be dysregulated as RA disease activity increases. Although only a few reports have reported a decrease in guanidoacetic acid level in disease, low homoarginine concentration is reported to be associated with myocardial dysfunction [28, 29] and renal failure [29, 30], and also affects the production of vasodilator nitric oxide (NO) and mineral metabolism [29]. As it is well-known that RA is sometimes comorbid with cardiovascular or renal diseases, dysregulation of the arginine metabolism pathway, represented by lower homoarginine and guanidoacetic acid in active patients, may be closely associated with the risk of these comorbidities.

Hypotaurine, another potential biomarker identified in urine, is reported to be involved in protection against oxidative stress [31]. Interestingly, we found in this study that several metabolites in the cysteine and methionine metabolism pathway, which is upstream of the taurine and hypotaurine metabolism pathway, is associated with RA disease activity. For example, cysteine and methionine in plasma positively and inversely correlated with DAS28-ESR, respectively (Table 3). Also, in urine, homoserine and gamma-glutamyl-2-aminobutyrate positively correlated with DAS28-ESR (Table 3), and ophthalmic acid was elevated in active RA patients (Table 4). These are also involved in the cysteine and methionine metabolism pathway. Furthermore, cysteine is known to be a component of the antioxidant glutathione and is involved in the transsulfuration pathway, which consists of interconversion of cysteine and homocysteine through the intermediate cystathionine [32]. Since some of the intermediates in this pathway correlate with DAS28-ESR, our study strongly suggests that the reverse transsulfuration pathway is actively induced as RA disease activity increases. Hydrogen sulfide (H_2_S), which is also produced from cysteine, is known as a signaling molecule that regulates the physiological process in inflammations [33, 34], and is reported to be increased in synovial fluids in RA patients [35]. Therefore, some pathways downstream of cysteine might be activated and the increase in urinary hypotaurine may represent these metabolic changes in active RA disease.

Taken together, we were able to discover the metabolites from plasma and urine that could be a combinatorial biomarker for RA. This finding supports the use of metabolomics analysis as a promising way to search for disease biomarkers, and to obtain deep insights into the disease pathophysiology, especially with multiple fluid/tissue samples. Metabolomics in combination with other omics methods, such as transcriptomics and proteomics, and the combination of the information obtained with that of other diseases would be beneficial for the better understanding of the state and course of individual RA patients such as with regard to the risk of comorbidity. However, confirming the validity of the biomarker candidates and the significance of the metabolic pathway in RA pathophysiology found in this study requires further study with a new and different set of samples and a larger sample size. We should also confirm whether or not the candidates were specific for RA, because we could not exclude the possibility that the metabolites correlated with the general inflammation process.

## Conclusions

We employed metabolic profiling using the CE-Q-TOFMS method to identify metabolites that were associated with disease activity in plasma and urine of patients with rheumatoid arthritis. As a result, we generated a list of metabolites that correlated significantly with DAS28-ESR, as well as metabolites that significantly differed between patients with active and inactive RA. Using both lists, three metabolites—histidine and guanidoacetic acid in plasma and hypotaurine in urine—were selected as MLR variables. Thus, this study demonstrates that the combination of metabolomics analysis of both plasma and urine samples is a useful approach to predicting biomarkers for RA and obtaining deep insights into the pathophysiology of this disease.

## Supporting information

S1 Dataset Metabolomics data with clinical data of RA patients and control subjects. (XLSX)

S1 Fig PCA score plot of control subjects and RA patients based on plasma metabolic profile. The green and red dots represent RA patient and control samples, respectively.
(TIF)

S2 Fig PLS-DA model validation by permutation tests (n = 1000) based on the plasma metabolic profile of RA patients and control samples. The p value was p = 0.022 (22/1000).
(TIF)

S3 Fig PCA score plot of active RA patients (n = 32) and inactive RA patients (n = 17) based on metabolic profiles in plasma and urine. The red and green dots represent samples of active patients (DAS28-ESR≥3.2) and inactive patients (DAS28-ESR<3.2), respectively.
(TIF)

S4 Fig PLS-DA score plot of active RA patients (n = 32) and inactive RA patients (n = 17) based on metabolic profiles in plasma and urine. The red and green dots represent samples of active patients (DAS28-ESR≥3.2) and inactive patients (DAS28-ESR<3.2), respectively. R^2^ = 0.95405 and Q^2^ = 0.12656 indicate that the model was overfitted.
(TIF)
