# Supplementary figures and images for: Metabolomic approach to the exploration of biomarkers associated with disease activity in rheumatoid arthritis
Source: PLoS One. 2019 Jul 11;14(7):e0219400. doi: 10.1371/journal.pone.0219400 (PMC6622493; doi:10.1371/journal.pone.0219400)

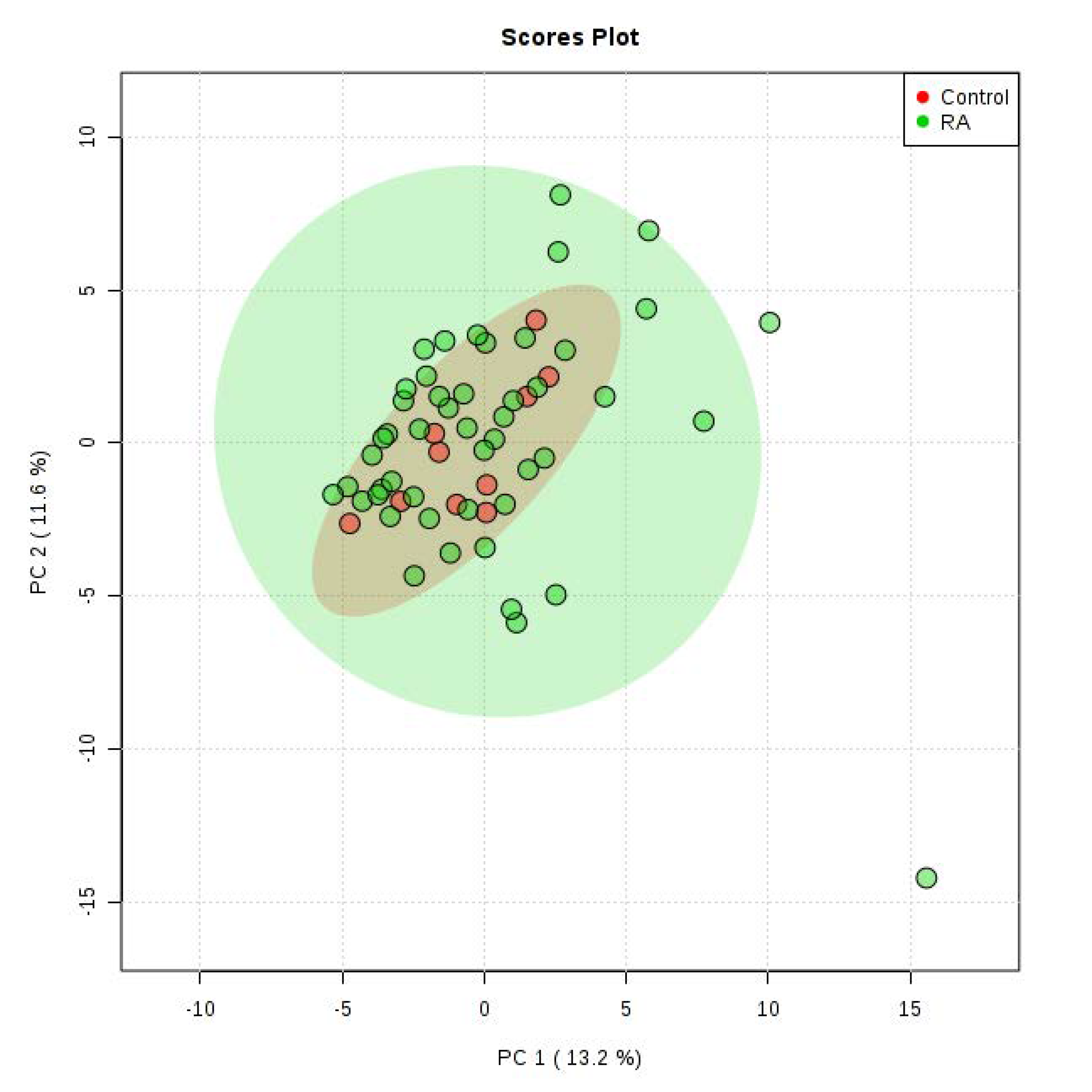

Supplement: S1 Fig — The green and red dots represent RA patient and control samples, respectively. (TIF) [file pone.0219400.s002.tif]

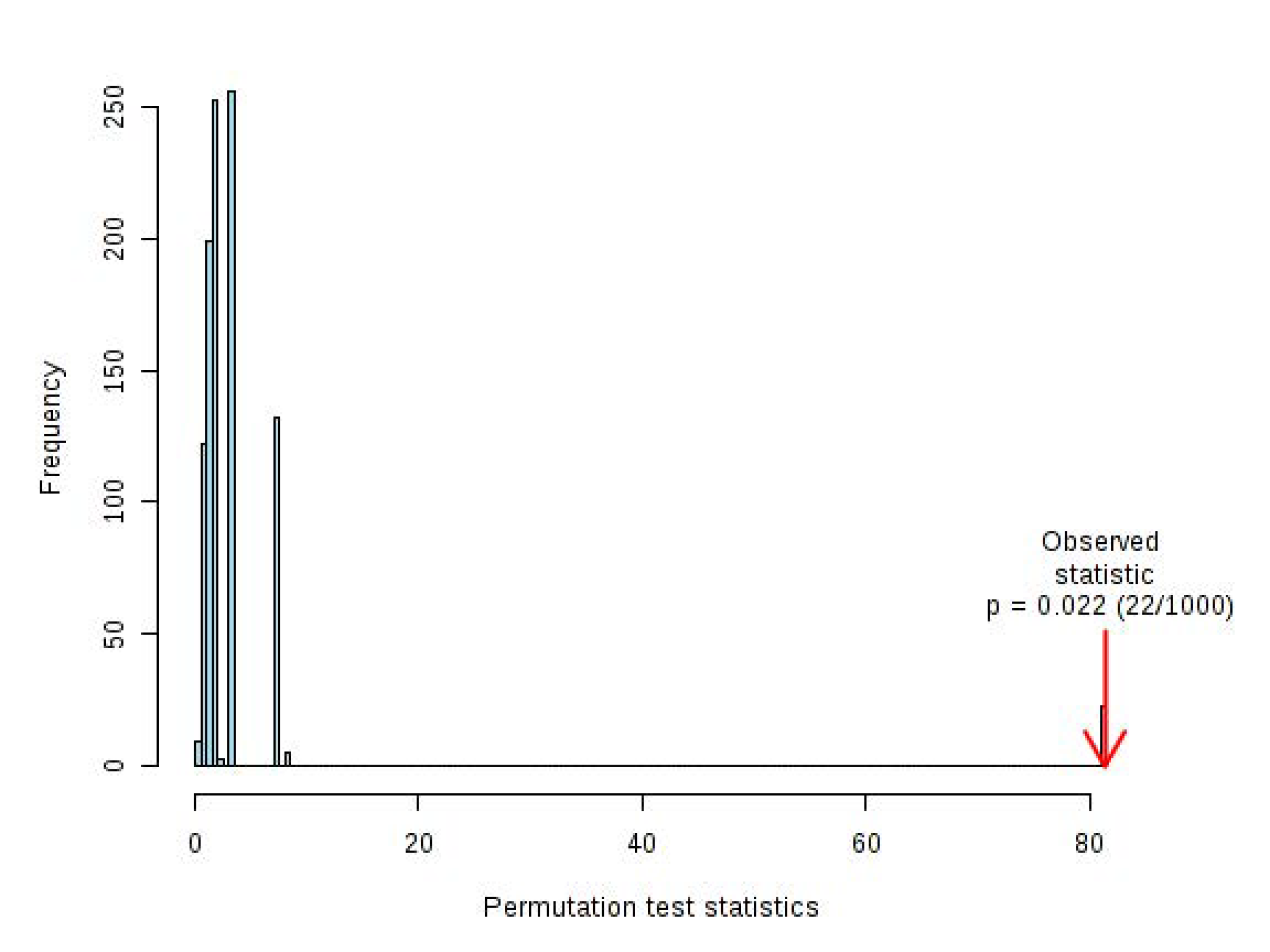

Supplement: S2 Fig — The p value was p = 0.022 (22/1000). (TIF) [file pone.0219400.s003.tif]

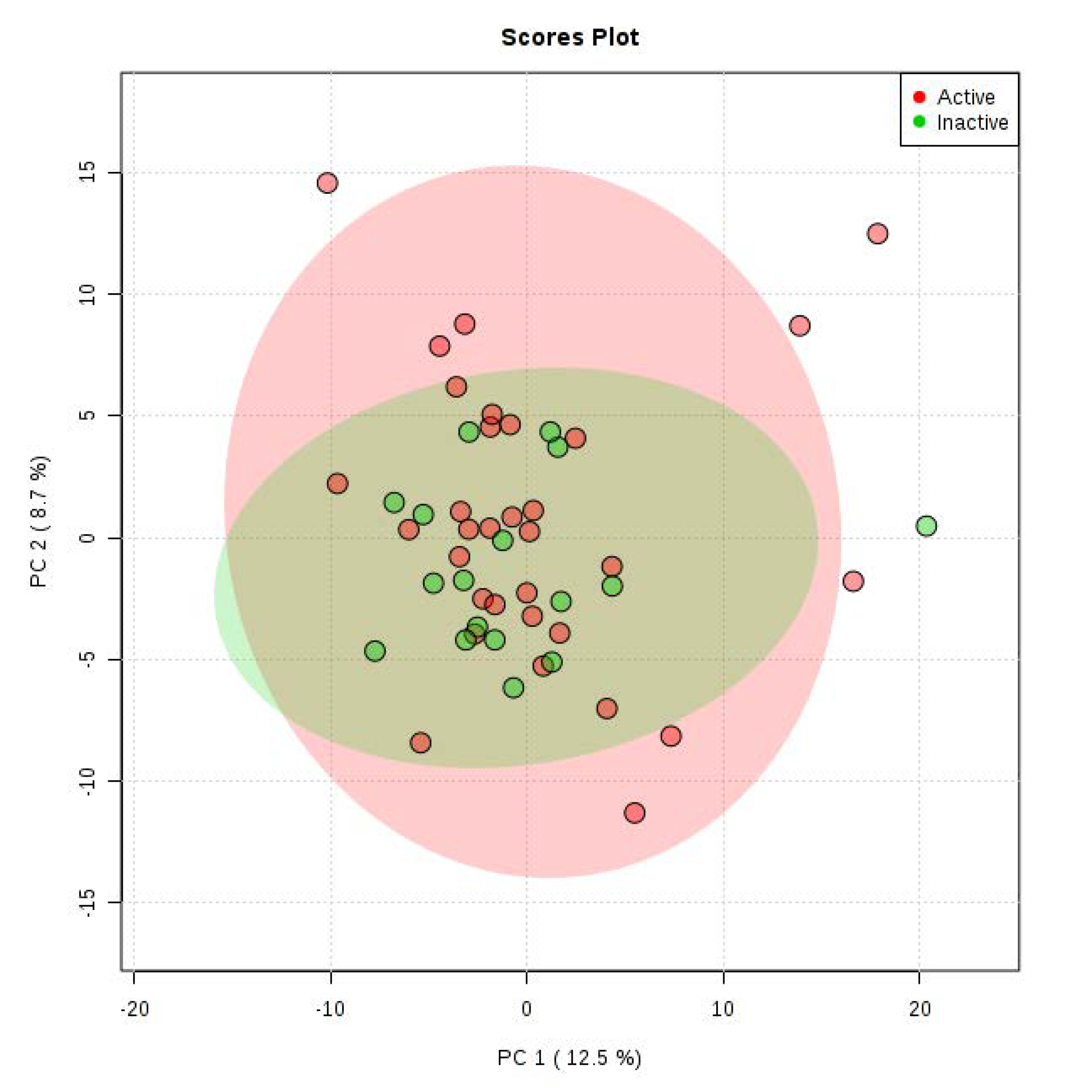

Supplement: S3 Fig — The red and green dots represent samples of active patients (DAS28-ESR≥3.2) and inactive patients (DAS28-ESR<3.2), respectively. (TIF) [file pone.0219400.s004.tif]

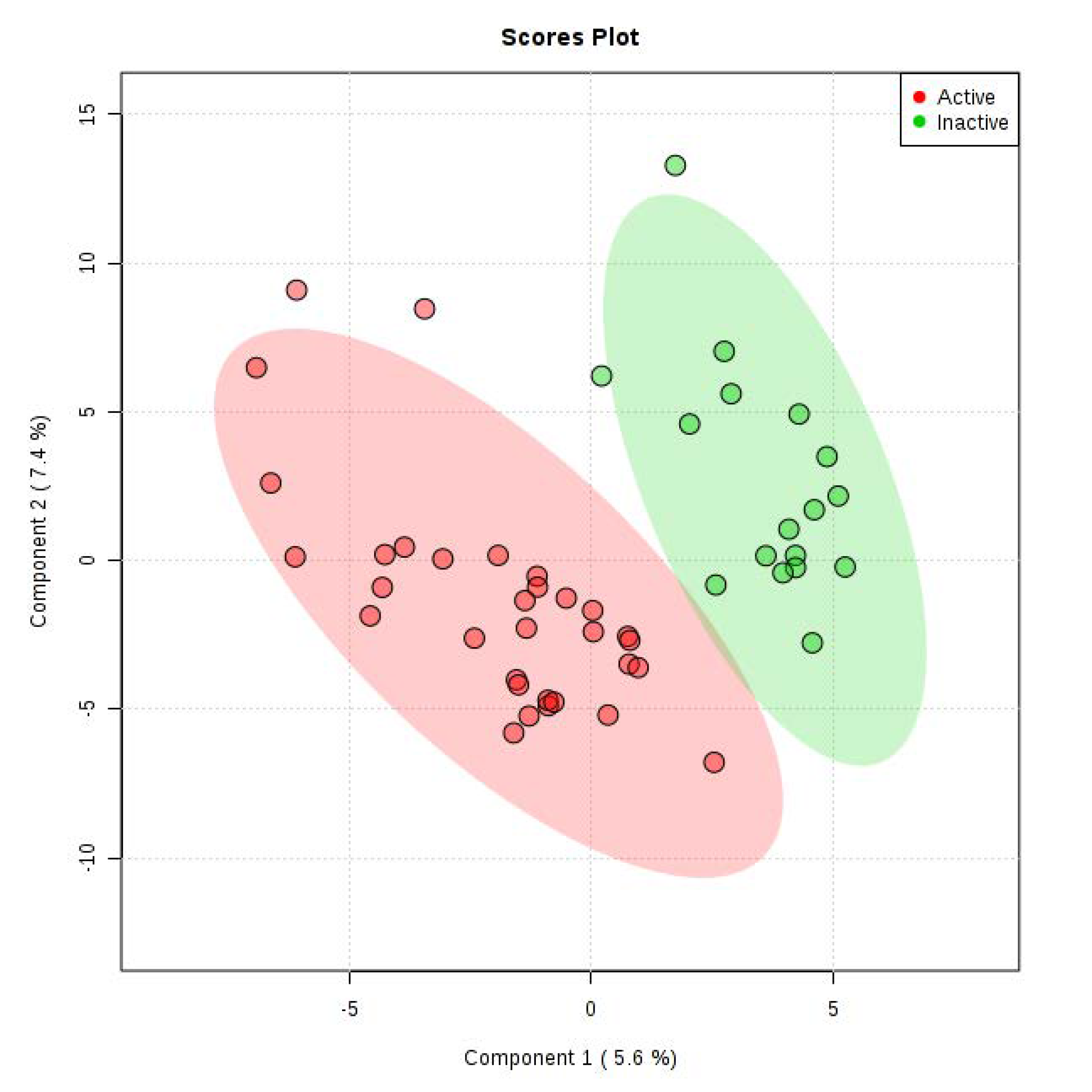

Supplement: S4 Fig — The red and green dots represent samples of active patients (DAS28-ESR≥3.2) and inactive patients (DAS28-ESR<3.2), respectively. R2 = 0.95405 and Q2 = 0.12656 indicate that the model was overfitted. (TIF) [file pone.0219400.s005.tif]
